# Restoration of RPGR expression in vivo using CRISPR/Cas9 gene editing

**DOI:** 10.1038/s41434-021-00258-6

**Published:** 2021-07-14

**Authors:** Jessica D. Gumerson, Amal Alsufyani, Wenhan Yu, Jingqi Lei, Xun Sun, Lijin Dong, Zhijian Wu, Tiansen Li

**Affiliations:** 1 Neurobiology Neurodegeneration & Repair Laboratory (N-NRL), National Eye Institute, Bethesda, MD USA; 2 King Saud bin Abdulaziz University for Health Sciences, Jeddah, Saudi Arabia; 3 Montgomery College, Rockville, MD USA; 4 Ocular Gene Therapy Core, National Eye Institute, Bethesda, MD USA; 5 Genetic Engineering Core, National Eye Institute, Bethesda, MD USA

**Keywords:** Gene expression, Genetic vectors

## Abstract

Mutations in the gene for Retinitis Pigmentosa GTPase Regulator (*RPGR*) cause the X-linked form of inherited retinal degeneration, and the majority are frameshift mutations in a highly repetitive, purine-rich region of *RPGR* known as the OFR15 exon. Truncation of the reading frame in this terminal exon ablates the functionally important C-terminal domain. We hypothesized that targeted excision in ORF15 by CRISPR/Cas9 and the ensuing repair by non-homologous end joining could restore *RPGR* reading frame in a portion of mutant photoreceptors thereby correcting gene function in vivo. We tested this hypothesis in the *rd9* mouse, a naturally occurring mutant line that carries a frameshift mutation in *RPGR*^ORF15^, through a combination of germline and somatic gene therapy approaches. In germline gene-edited *rd9* mice, probing with RPGR domain-specific antibodies demonstrated expression of full length RPGR^ORF15^ protein. Hallmark features of RPGR mutation-associated early disease phenotypes, such as mislocalization of cone opsins, were no longer present. Subretinal injections of the same guide RNA (sgRNA) carried in AAV sgRNA and SpCas9 expression vectors restored reading frame of *RPGR*^ORF15^ in a subpopulation of cells with broad distribution throughout the retina, confirming successful correction of the mutation. These data suggest that a simplified form of genome editing mediated by CRISPR, as described here, could be further developed to repair *RPGR*^ORF15^ mutations in vivo.

## Introduction

Retinitis pigmentosa (RP) is a heterogenous group of inherited retinal diseases caused by a progressive degeneration of rod and cone photoreceptors that results in the eventual loss of vision. Mutations in more than 200 genes cause RP and collectively these affect one in 3000–5000 people, representing a major cause of inherited forms of blindness globally. Mutations causing X-linked RP (XLRP) are particularly severe with retinal disease presenting within the first few decades of life. The majority of mutations causing XLRP have been identified as loss-of-function alleles in the gene encoding retinitis pigmentosa GTPase regulator (*RPGR*) [1–4].

The RPGR protein is located within the photoreceptor connecting cilium [5], a membrane-enclosed microtubule-based structure analogous to the transition zone of primary cilia that links the biosynthetic photoreceptor inner segment and the light sensing outer segment. The outer segment is a specialized compartment packed with stacks of membranous discs containing the phototransduction machinery required for high sensitivity to visual stimuli. The inner segment contains the bulk of cytoplasm and organelles and is thus the site of core cellular processes such as energy production and protein synthesis. Because the distal ends of outer segments are continuously shed and phagocytosed by apposing retinal pigment epithelia (RPE), phototransduction proteins must be continuously produced in the inner segment and trafficked across the connecting cilium. This process is highly regulated and involves several proteins associated with retinal disease, including RPGR, RPGRIP1, and CEP290 [6]. In the absence of RPGR function, ciliary gating appears compromised and proteins may move more freely between compartments, disrupting cell homeostasis and consequently leading to cell death. The abnormal accumulation of opsins in the cell bodies of RPGR-deficient cone photoreceptors is an early and robust phenotype [5, 7, 8]. Similar phenotypes are observed in other animal models where connecting cilia proteins are mutated or absent [9–11].

Alternative splicing of *RPGR* generates multiple transcripts, and two major isoforms are well characterized [4, 12]. The default isoform, *RPGR*^ex1–19^, is broadly expressed in multiple tissues, whereas a tissue-restricted *RPGR*^ORF15^ is expressed primarily in retinal photoreceptors. The *RPGR*^ORF15^ variant contains a unique 3ʹ terminal ORF15 exon that encodes two critical functional domains: a highly repetitive glycine/glutamic acid-rich domain and a C-terminal basic domain with homology to tubulins. The length of the repetitive region varies greatly among species and even among healthy human individuals. Furthermore, in-frame deletions that shorten this region by up to one third are well tolerated and appear to cause no functional deficits in mice [13, 14]. Nevertheless, a sufficiently high number of glutamic acid residues in this region need to be preserved for glutamylation, a post-translational modification essential for RPGR function and the absence of which causes retinal disease [15]. In contrast, mutations that disrupt the reading frame and ablate the C-terminal domain invariably compromise protein function and cause retinal disease. The ORF15 exon harbors more than 70% of known disease-causing mutations in *RPGR* making it an attractive target for gene therapy [4, 16].

While gene replacement therapies for RPGR-associated diseases have shown promise as a therapeutic strategy [17–21], engineering and delivering recombinant *RPGR* gene constructs are fraught with elevated risks due to the unique characteristics of the sequence. The repetitive region of RPGR is inherently unstable, difficult to sequence, and prone to spontaneous mutations during in vitro handling and subcloning [20]. Wildtype *RPGR* genes packaged into AAV vectors have later been found to contain many spontaneous changes in this repetitive region [21]. Moreover, overexpression of RPGR can be toxic and requires careful dosage for gene delivery [21]. To further improve the prospect for successful gene therapies, we sought to test an alternative strategy based on a simplified form of genome editing, whereby a gene mutation is corrected in the chromosome in situ. For any disease-causing gene mutation, gene editing offers the exciting prospect of the ultimate cure. This approach employs the CRISPR/Cas9 system to introduce a double-stranded break (DSB) first at a target site followed by template-dependent, homology-directed repair, which swaps out the mutant with the wildtype sequence. With the current technology, the first step is highly efficient in disrupting gene function in vivo [22] but the second step is rate-limiting due to its low efficiency. Genome editing based on this approach has been validated in cell lines cultured in vitro, in which low frequency rescue event can be selected for and amplified but has not met with much success in vivo. In the absence of template-mediated homologous recombination, a CRISPR-mediated double strand break is followed by non-homologous end joining (NHEJ), a host cell repair mechanism that usually results in the insertion or deletion of base pairs. CRISPR-based gene editing has been explored as a potential gene therapy for inherited retinal degeneration [23, 24], and can be useful to disrupt dominant mutant alleles [25–27]. Recent studies have also shown that *RPGR*^ORF15^ can be targeted via CRISPR/Cas9 in induced pluripotent stem cells [28, 29]. Correcting mutations through genome editing in vivo is, however, currently not feasible for most genes.

Taking advantage of the unique features of the *RPGR* OFR15 exon, we have proposed a simplified, variant version of gene editing. In this design, a double-stranded break in genomic DNA with CRISPR is first introduced at a target site in the repetitive region. Then the ensuing NHEJ would produce random base insertions or deletions that could in theory shift the reading frame in up to one third of cells thereby restoring expression of the critical C-terminal domain. While the therapy is not expected to restore gene structure in all target cells, based on previous clinical studies of retinal degenerative conditions and the known reserve capacity in retinal neurons, even a small fraction of surviving photoreceptors will support meaningful visual function and help maintain quality of life for the affected individuals. This design obviates the need for the rate-limiting and template-dependent homologous recombination step making it potentially feasible for in vivo intervention. As a proof-of-principle to treat XLRP caused by RPGR frameshift mutations, we chose the naturally occurring *rd9* mouse model, which carries a 32-bp insertion in the ORF15 exon resulting in a frame shift that truncates RPGR^ORF15^ protein [7, 30]. We tested this novel form of gene editing in *rd9* mice using CRISPR, delivered via subretinal injection of AAV vectors or pronuclear injection of sgRNA/SpCas9 ribonucleoprotein (RNP) complex, to restore RPGR function in somatic retinal and germline cells. We examined whether this approach could restore the full-length RPGR^ORF15^ expression and alleviate the disease phenotype.

## Materials and methods

### Animal use

All procedures performed with animals were approved by the Animal Care and Use Committees at the National Institutes of Health and in accordance with the ARVO Statement for the Use of Animals in Ophthalmic and Vision Research. Both male and female *rd9* and C57BL/6 mice were used and for all experiments, *rd9* littermates or age matched C57BL/6 litters were used for comparisons. Details regarding the age, number of animals analyzed, and blinding conditions are described in the respective figure and/or legend. Unless otherwise noted, animals were euthanized and tissue collected at 3–6 weeks of age.

### Germline editing of the *rd9* allele in mice

The sgRNA target sequence (5ʹ-GAAGAGGGGGAGAGGAAGAA-3ʹ) was selected using the CRISPR design tool (http://crispr.mit.edu/) and Benchling (https://benchling.com/). Several other sgRNAs were tested in the germline editing experiments but proved unsatisfactory. The sgRNA was synthesized with T7 in vitro transcription as described [31], and mixed with SpCas9 protein (PNA Bio) to form gRNA-Cas9 ribonucleoprotein (RNP) particles [32]. Cas9/gRNA ratios between 50 ng/10 ng and 150 ng/50 ng were tested, and the best performing ratio was found at 50 ng Cas9 protein with 10 ng guide RNA. The RNP particles were microinjected into zygotes of *rd9* mice (*rd9/rd9*) as described [33]. Litters of F0 founders were born and screened by PCR and sequencing for potential genomic changes.

### Immunohistochemistry

Following euthanasia, eyes were removed and either frozen immediately in embedding media (either OCT or M1) or fixed in 4% paraformaldehyde/PBS. For samples in fixative, the anterior segment was removed after 5–10 min of fixation and the posterior eye cup was returned to fixative for several hours. Eye cups were then washed several times in PBS, embedded in 7% Type XI agarose, and cut into 75–100 μM cross sections using a vibratome. Frozen eyes were sectioned using a cryostat, collected on slides, and subsequently fixed in 1–4% paraformaldehyde immediately prior to antibody staining. All sections were blocked in 5% donkey serum (Equitech-Bio) in PBS-T for >1 h prior to incubation with antibodies diluted in blocking buffer. After overnight incubation in primary antibody, sections were washed several times in PBS before incubation with secondary antibody and DAPI for 1–4 h. Following several washes with PBS, sections were mounted in FluormountG (Southern Biotech). Slides were imaged on either a Zeiss LSM 700 or 880 confocal microscope and processed using Zen software. Maximum intensity projections were generated from z-stack images representing 2–8 μM of section thickness and for each dataset, the number of slices and imaging plane thickness were kept constant between treatment groups.

### Immunoblot analysis

Following euthanasia eyes were removed, and the retina was dissected from the eye and snap frozen on dry ice. Single retinas (*n* = 3 per genotype) were homogenized independently in RIPA buffer (150 mM NaCl, 50 nM Tris, pH 8.0, 1% IGEPAL, 0.5% Deoxycholate, 0.1% SDS) containing 1X protease inhibitors (Roche). Lysate concentration was determined using a Pierce BCA protein assay kit (Thermo) and samples were equally loaded and separated on a 4–15% TGX Stain-Free SDS-PAGE gel (Bio-Rad). Following electrophoresis, separated proteins were wet transferred to PVDF for immunoblotting. Blots were incubated in StartingBlock PBS blocking buffer (Thermo) for >1 hr prior to incubation with antibodies diluted in blocking buffer. After overnight incubation in primary antibody, blots were washed several times in PBS before incubation with secondary antibody for approximately 1 h. After several PBS washes, blots were incubated with substrate (SuperSignal West Pico, Thermo) and digitally imaged on a Bio-Rad ChemiDoc imaging system.

### Antibodies

Antibodies used that were custom generated in our laboratory included RPGR-s1 (rabbit), RPGR-570 (rabbit), and c100 (rabbit), S-Opsin (chicken), and Rootletin (chicken). Other antibodies included Rhodopsin 1D4 (mouse; Gift from Bob Molday), M-Opsin (rabbit; Millipore AB5405), GT335 (mouse; Adipogen AG-20B-0020), AHI1 (mouse; Abcam ab93386), and Glial Fibrillary Acidic Protein (mouse; Sigma G3893).

### AAV production

Dual AAV vectors were generated: one containing a Streptococcus pyogenes Cas9 (SpCas9) driven by the rhodopsin kinase (RK) promoter [34] and another containing both the RPGR-targeted sgRNA (see above) driven by the U6 promoter and tdTomato reporter gene driven by RK promoter. Detailed methods for AAV generation and CRISPR/Cas9 genome editing via AAV in mouse retina have been recently published [35].

### Subretinal injections

Mice (*n* > 18) were anesthetized via IP injection of ketamine/xylazine and eyes were dilated and topically anesthetized via drops of 0.5–1% tropicamide, 2.5% phenylephrine hydrochloride, and 0.5% proparacaine. A small incision was made through the cornea anterior to the limbus and a blunt needle fitted to a Hamilton syringe was inserted to deliver ~1 μl of each AAV (7.5e9–1e10 vg/μl) diluted in PBS/fluorescein, to the subretinal space. Animals were euthanized 10–12 weeks post injection and the eyes collected for analysis. A larger sample size was chosen for analysis based on the variability observed in preliminary dose–response experiments. Because each mouse received both RPGR-targeted (right eye) and control non-targeted AAV (left-eye), treatment group randomization was not necessary.

### Amplification and sequencing of RPGR^ORF15^

Genomic DNA isolated from either tail biopsy or retina was used as a template to amplify a region of *RPGR-ORF15* spanning the 32-bp insertion in *rd9* mice. Rescue was confirmed in AAV-treated retinas via immunohistochemistry of frozen sections, and the remaining retina was dissected from the frozen block and used for DNA extraction. Two forward primers and one reverse primer were used for PCR, F1: 5ʹ-agaggaagagggggaaggcgaggg-3ʹ, F2: 5ʹ- gggtggaaggaaggagaggagcaagaac-3ʹ, and R1: 5ʹ-ccacatcatcctcacaacttccgtgtt-3ʹ. PCR was performed using PrimerSTAR HS with GC buffer (Takara/Clontech) using the following thermocycling conditions: one cycle of 98 degrees for 2 min; 35–40 cycles of 98 degrees for 10 sec, 68 degrees for 45 sec; and a final cycle of 68 degrees for 1 min. PCR product was sequenced by Genewiz using either Sanger or PacBio deep sequencing platforms. For PacBio sequencing, the PCR amplicon was generated from a pool of three retinas from either RPGR-targeted or non-targeted (GFP) AAV-injection. CCS analysis of the single pass non-consensus raw data was performed by Genewiz. Returned reads were further filtered in Matlab 9.4 for alignment to a reference *rd9* ORF15 sequence.

## Results

### Restoration of full-length RPGR^ORF15^ following CRISPR-mediated gene editing

**Fig. 1:**
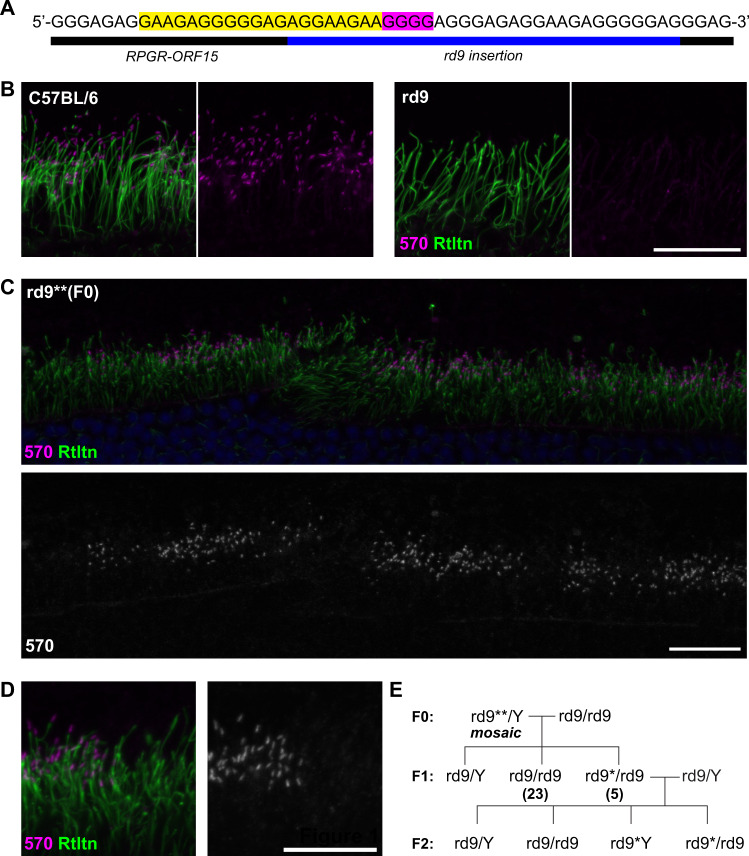
Germline correction of RPGR-ORF15. **A** Schematic showing the target region of ORF15 sequence in *rd9*. The sequence complementary to the sgRNA is highlighted in yellow and the PAM sequence highlighted in magenta. **B** Confocal images showing expression of RPGR-ORF15 protein at the connecting cilia in control (C57BL/6) retina using the ORF15-specific antibody, RPGR-570. RPGR-ORF15 is absent in *rd9*. Ciliary rootlet stained with Rootletin (Rtltn). **C** Confocal images of one *rd9* founder (rd9**) following CRISPR/Cas9 modification showing mosaic expression of RPGR-ORF15. **D** High magnification image of the same retina shown in (**C**) demonstrating RPGR-ORF15 localization at the connecting cilium. **E** Breeding strategy used to separate CRISPR-modified alleles from founder (rd9**/Y). For the F1 generation, numbers in parentheses indicate the number of females analyzed for each genotype. Scale bars in (**B**), (**C**), and (**D**) indicate 20 μm.

To demonstrate the feasibility of our approach, we first used germline delivered CRISPR/Cas9 to correct the *rd9* mutant allele in mice by injecting the constructs into fertilized mouse embryos. A 20-bp target sequence was selected for the sgRNA that included part of the native ORF15 sequence and the first 8 bp of the mutant duplication in *rd9* (Fig. 1A). The resulting F0 mice were screened for genome editing events via genomic PCR, and a single founder was identified from multiple batches of microinjections. The yield was much lower than the typical yield of founders following similar CRISPR/Cas9 microinjections for other targeted genes in our laboratory, both in terms of live offspring born and founder mice with genome modifications. The reason for the lower yield remains unclear but could be related to potential toxicity of the guide RNA sequences, which were highly repetitive and contained only purine bases. Founders were also screened later via immunofluorescence staining of the retina for in-frame RPGR^ORF15^ protein to detect productive genome editing events. An RPGR^ORF15^ isoform-specific antibody, RPGR-570, detected RPGR^ORF15^ at the connecting cilia in control C57BL/6 but not in *rd9* mouse retinas (Fig. 1B). In the founder that carried a gene-edited allele (rd9**), RPGR^ORF15^ protein was observed in patches of photoreceptors throughout the retina (Fig. 1C) indicating a mosaic retina with some, but not all, photoreceptors having undergone a correct genome editing event. Mosaicism in transgenic founder mice produced by pronuclear microinjection of DNAs is a common phenomenon and an expected outcome with our approach. Higher magnification of stained retinas (Fig. 1D) showed localization of RPGR^ORF15^ at the connecting cilia in clusters of neighboring photoreceptors. The morphology, positioning, and signal intensity of labeled connecting cilia are indistinguishable from those of control retinas. The founder was backcrossed to the parental *rd9* strain, and screening of F1 females (only female offspring could have inherited the X-linked gene) further demonstrated germline mosaicism of the founders with ≈18% of female progeny receiving the edited allele (designated as rd9*; Fig. 1E).

**Fig. 2:**
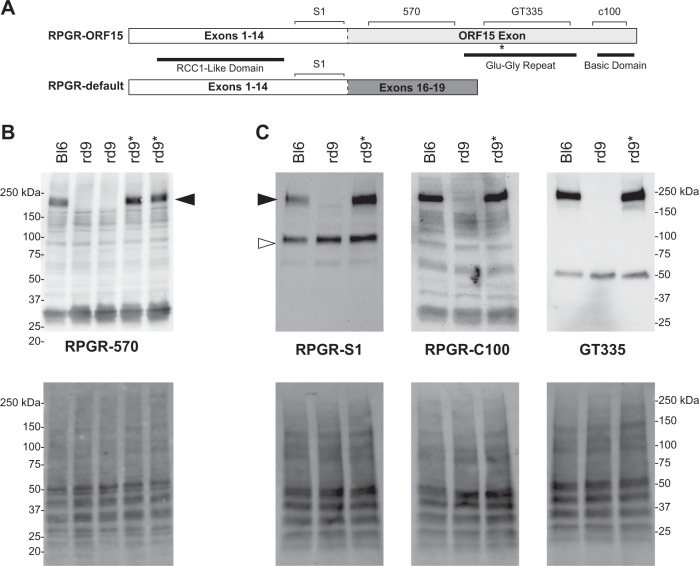
Correction of C-terminus in rd9* modified mice. **A** Cartoon depicting both protein isoforms of RPGR expressed in the retina and the epitopes recognized by the antibodies used in the study. The dashed line identifies where the two proteins differ as a result of alternative splicing. **B**, **C** Western blots of retinal lysates from C57BL/6 (Bl6), naïve *rd9*, and CRISPR-modified (rd9*) mice (upper panels). Lower panel images show the same blot imaged for total protein loaded. **B** RPGR-570 antibody detects only the RPGR-ORF15 isoform (black arrowheads) in Bl6 and rd9* mice. **C** RPGR-s1 antibody (left panels) detects both RPGR-ORF15 and RPGR-default (white arrowhead) in all mice except *rd9*, which lacks RPGR-ORF15. RPGR-c100 antibody (middle panels) detects the far c-terminus of RPGR-ORF15 in both Bl6 and rd9*. GT335 antibody (right panels) detects post-translationally modified RPGR-ORF15 in both Bl6 and rd9*. Modified tubulin is also observed at ~50 kDa.

Western blots performed on retinal lysates from F2 and later generations of mice showed that RPGR^ORF15^ protein from homozygous (female) and hemizygous rd9* (male) mice was expressed at levels comparable to controls (Fig. 2B). Additional RPGR-specific antibodies were tested that recognize different functional domains of RPGR^ORF15^ (Fig. 2A). Whereas *rd9* mice expressed only the RPGR^ex1-19^ protein as detected by the RPGR-S1 antibody, both isoforms were expressed in C57BL/6 control and gene-edited rd9* mice (Fig. 2C). A truncated RPGR^ORF15^ from the mutant allele was not detected by either the RPGR-S1 or RPGR-570 antibodies, consistent with the truncated protein being unstable [7]. The repetitive Glu–Gly domain in RPGR^ORF15^ is glutamylated, a post-translational modification that is essential for RPGR function in photoreceptors [15]. Antibodies that detect the RPGR-C-terminus and glutamylated RPGR (C100 and GT335, respectively; Fig. 2C) also showed similar reactivity between control and rd9* retina, demonstrating that RPGR^ORF15^ from the genome-edited rd9* allele was likely to be functional.

### Rescue of the disease phenotype in photoreceptors

**Fig. 3:**
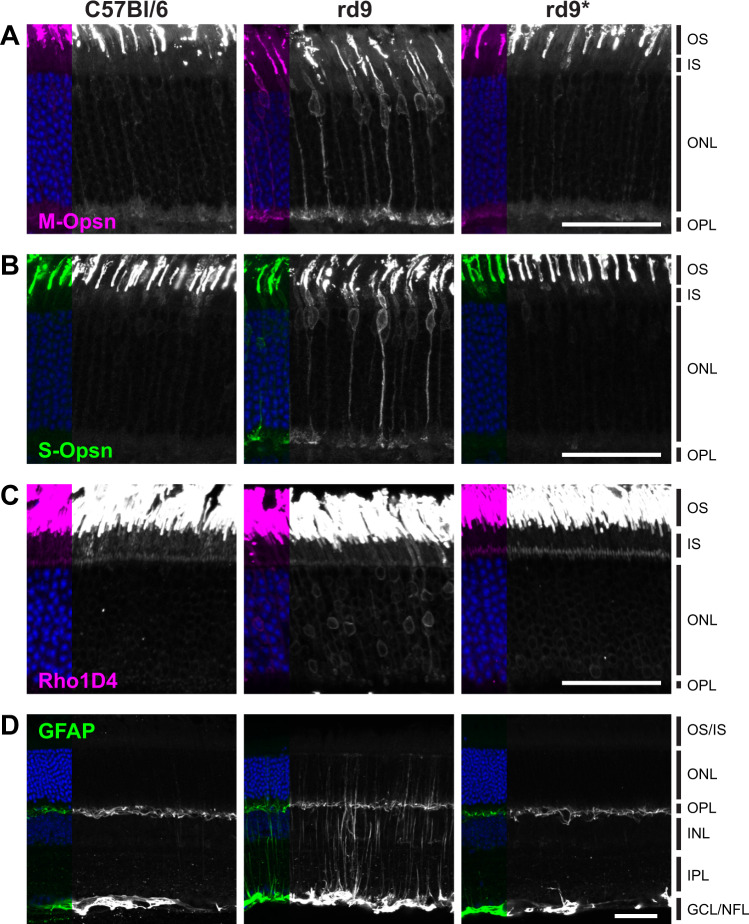
Correction of *rd9* retinal pathology in germline treated mice. Confocal images of retina from control C57BL/6 (Bl6) (*n* = 2), naïve *rd9* (*n* > 3), and CRISPR-treated (rd9*) mice (*n* > 3)**. A**, **B** Cone opsins (M-opsn, S-Opsn) are mislocalized throughout cone photoreceptors in young *rd9* retina but correctly localized to the outer segments (OS) in both Bl6 and rd9* retina. **C** Rhodopsin (Rho1D4) is also mislocalized throughout rod photoreceptors in older (≈8 months) *rd9* retina but expressed normally in Bl6 and rd9* retina. **D** Activated Muller glia, as detected by GFAP, are identified in *rd9* retinas but in Bl6 and rd9* tissue, expression is restricted to the outer plexiform (OPL) and nerve fiber layers (NFL). DAPI was stained in all images but only shown on the left of each panel. All scale bars indicate 50 μm. Inner segments (IS), outer nuclear layer (ONL), inner nuclear layer (INL), inner plexiform layer (IPL), and ganglion cell layer (GCL) also labeled for reference. All *rd9* and rd9* retinas were processed and imaged blind.

Although mice lacking RPGR demonstrate a slower progression of retinal disease than human patients, early mislocalization of cone opsin is a hallmark feature of the disease in mice. Glial activation, as revealed by GFAP upregulation, also occurs early indicating that photoreceptors are already under stress even though cell loss is not yet apparent. Both M-Opsin (Fig. 3A) and S-Opsin (Fig. 3B) can be detected throughout the cell body and synapse of *rd9* cone photoreceptors. However, in rd9* mice, cone opsin localization was confined to the outer segments, similar to C57BL/6 controls. By 8 months, Rhodopsin mislocalization is also elevated throughout rod photoreceptors of *rd9* mice (Fig. 3C), and many activated Muller glia are present (Fig. 3D). In contrast, retinas from rd9* mice appeared healthy and were indistinguishable from C57BL/6 controls. These data demonstrate phenotypic reversal of mutant photoreceptors following genome editing.

### Restoration of RPGR^ORF15^ in subpopulation of photoreceptors following subretinal delivery of AAV-CRISPR/Cas9

**Fig. 4:**
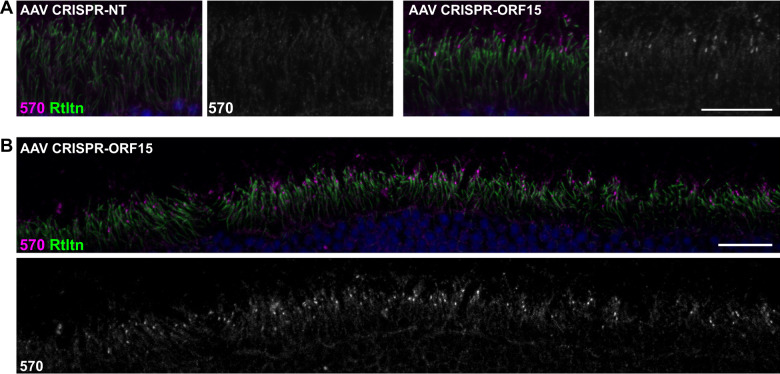
Correction of RPGR-ORF15 in AAV-treated *rd9* mice. **A** Confocal images of representative AAV-treated *rd9* retinal cross sections demonstrating rescue. Expression of RPGR-ORF15 is observed only in ORF15-targeted (AAV CRISPR-ORF15) but not non-targeted (AAV CRISPR-NT) retina, as indicated by a subset of cells positive for RPGR-570 signal at the connecting cilium. The ciliary rootlet is stained with Rootletin (Rtltn). Right panels show desaturated 570 channel to better visualize positive signal. **B** Lower magnification image of staining depicted in panel (**A**) to better show broad distribution of correction in treated retina. Lower panel shows desaturated 570 channel. Scale bars indicate 20 μm. Injections were performed in 3–6 week old *rd9* mice, and tissues were collected and analyzed 8–12 weeks post injection.

**Fig. 5:**
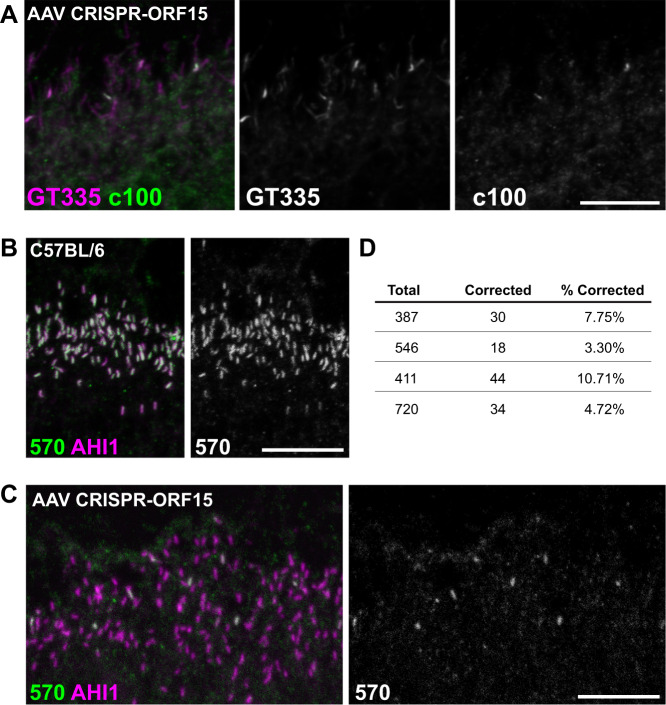
Complete correction of RPGR-ORF15 in AAV-treated mice. **A** Representative confocal images of CRISPR-targeted ORF15 (AAV CRISPR-ORF15) *rd9* retinal cross sections. Staining with additional RPGR antibodies c100 and GT335 confirm RPGR-ORF15 expression in treated retina. **B** Confocal images of control C57Bl/6 retinal cross sections stained with RPGR-570 and AHI1 to demonstrate the assay used to quantify rescue percentages. In healthy control retina, RPGR-ORF15 (570) and AHI1 are near perfectly colocalized at the connecting cilium. **C** RPGR-570 and AHI1 staining in AAV CRISPR ORF15 treated *rd9* retinal cross sections. All connecting cilia are positive for AHI1 and a subset are also positive for 570 indicating correction of RPGR-ORF15 in these cells **D** Quantification of rescue in four large fields of AAV CRISPR ORF15 treated retina. Injections were performed in 3–6 week old *rd9* mice, and tissues were collected and analyzed 8–12 weeks post injection.

Having validated our genome editing design in germline experiments, we next sought to investigate if the same strategy could be effective in correcting the *rd9* mutation in mature photoreceptors as a “proof-of-concept” for a potential therapy for *RPGR*^*ORF15*^ mutations. The same sgRNA for SpCas9 was cloned and packaged into AAV2/8 vectors and subretinally injected into *rd9* mice. We chose the well-established rhodopsin kinase (RK) promoter [36] to drive expression of Cas9 and a U6 *rd9* ORF15 sgRNA that also contained a tdTomato reporter. An irrelevant target sgRNA (GFP) was used as a negative control. Subretinal injections were performed in *rd9* mice and tissues were collected 8–12 weeks post injection for analysis. Confocal images of RPGR-570 antibody co-stained with a marker to the ciliary rootlet demonstrated expression of RPGR^ORF15^ at the connecting cilium in a subset of ORF15-treated photoceptors (Fig. 4A, right panels). Lower magnification images showed that RPGR^ORF15^ expression was broadly distributed throughout the retina (Fig. 4B). Additional markers for domains unique to the RPGR^ORF15^ isoform were also observed in treated retinas. The detection of glutamylation and the C-terminal domain suggested that RPGR protein was full length and thus likely functional (Fig. 5A). The efficiency of correction was determined by co-labeling treated retina sections with the ciliary marker AHI1, which labels all photoreceptors and colocalizes with RPGR in control retina (Fig. 5B). Images were taken in regions of the retina where patches of RPGR staining were observed (i.e., near the injection site), and AHI1 and RPGR-570 antibody positive cells were quantified (Fig. 5C). The number of dual-labeled photoreceptor cells ranged from 3.3–10.7% (Fig. 5D), which represented the percentage of photoreceptors having undergone a correct genome editing event that restored the reading frame of the *RPGR* gene.

### Identification of sequence changes in RPGR-ORF15 mediated by CRISPR/Cas9

**Fig. 6:**
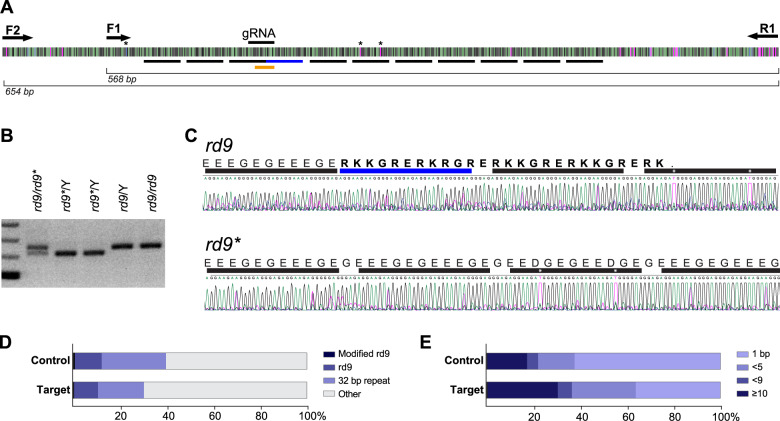
RPGR-ORF15 sequencing following CRISPR modification. **A** Schematic of the repetitive ORF15 sequence and primers used for PCR amplicon sequencing. Underlined black bars indicate locations of the 32-bp repeats and the underlined blue bar indicates the insertion in the *rd9* mutant. Gene modifications were identified by mismatch within the region underlined in orange. Asterisks indicate unique base pairs used for alignments. **B** PCR strategy used to identify and sequence germline modified RPGR-ORF15 (rd9*). Amplification of the ORF15 locus in rd9* mice demonstrated a small deletion, evidenced by a smaller molecular weight band. **C** Sanger sequencing of the rd9* amplicon shown in (**B**). Protein coding sequence shown to highlight the restored reading frame in rd9* mice. Bars and asterisks are analogous to those depicted in panel (**A**). Correction in rd9* was identified as a precise 32-bp deletion**. D** Analysis of reads from PacBio deep sequencing of AAV ORF15-targeted retina and non-targeted control retina. Most sequences were degenerate with only 20–40% containing at least one precise 32-bp repeat (32 bp repeat). Only 11–12% of reads could be unambiguously mapped to the *rd9* insertion region but very few (64 control; 33 ORF15 target) were identified as modified from native *rd9* sequence (Modified rd9). The identification of modifications in both ORF15 and control-treated retina suggests these are sequencing errors. **E** Analysis of INDEL lengths identified in panel (**D**).

Having confirmed the restoration of the open reading frame in the ORF15 exon of rd9* treated retinas, we next sought to identify the specific sequence changes that had occurred after CRISPR-mediated editing. Due to the well-known difficulty in manipulating RPGR^ORF15^ [13], several polymerases and primers were initially tested and a primer set (see Methods) was identified that amplified up to 690 bases flanking the repetitive CRISPR-targeted region (Fig. 6A). To determine the edited gene sequences resulting from germline modification, PCR was performed on genomic DNA extracted from F2 offspring. A downward shift in the molecular weight was observed indicating that the modifications were likely to be deletions (Fig. 6B). Sanger sequencing of the rd9* amplicon revealed that a 32-bp sequence was deleted from the *rd9* allele thus restoring the open reading frame through this region (Fig. 6C). Surprisingly, deletion of 32-bp in this region would result in a sequence that is indistinguishable from that of C57BL/6. Because the region is highly repetitive, it was difficult to locate precisely the sequence that was deleted. However, two thymidine residues within the fourth 32-bp repeat and a unique 5ʹ cytosine (Fig. 6A, asterisks) were used to align the amplicon sequence and confirm that the original *rd9* insertion had been eliminated. Because a complete reversion of the mutant allele to wildtype was unexpected, we conducted further rounds of embryo injections to generate additional independent founders. Of more than 30 additional F0 mice screened via genomic PCR and detection with the RPGR-570 antibody, only one additional founder was identified that exhibited mosaic RPGR^ORF15^ expression in the retina. Sanger sequencing was again performed and unexpectedly, the genomic modification in the second founder was identical to the first one. Because the founders were generated from independent experiments, these results were not consistent with NHEJ, which is thought to be largely random in nature, and strongly suggest a bias in the sequence repair of DNA breaks in the purine-rich repetitive region *RPGR*^*ORF15*^.

To identify gene editing events following AAV-mediated retinal delivery of CRISPR/Cas9 and to determine whether 32-bp deletions were common occurrences, genomic DNA was isolated from AAV-treated retinas and subjected to deep sequencing. High fidelity PCR was performed to generate amplicons that were subsequently purified. Due to read length limitations with Illumina-based sequencing and the highly repetitive nature of the amplicons, resulting PCR products were sequenced using the PacBio platform. Previous Sanger sequencing of the *rd9* allele was used to generate a reference sequence to identify genomic changes in AAV-treated retinas. The PacBio reads were highly heterogenous and divergent from *rd9* sequence with only 30-40% of sequences (6,657/16,783 control; 5,268/17,455 ORF15 target) containing a single precise 32-bp repeat of the ORF15 repetitive region (Fig. 6D). Of those, roughly a third contained enough unique sequence in flanking regions to map unambiguously to the *rd9* insertion (Fig. 6A, blue bar) and determine whether genome modification occurred. In both the ORF15-targeted and control samples, many mismatches were identified throughout ORF15, most frequently single base changes within strings of polyA or polyG, suggesting artifacts in either the amplification or downstream sequencing. Nevertheless, we sought to identify genuine CRISPR modifications by filtering for mismatches within a 14-bp sequence spanning the *rd9* insertion (Fig. 6A, orange bar). Filtering resulted in <100 reads that were subsequently manually verified. Changes observed in the ORF15-targeted samples were also present in the control samples (Fig. 6D, E), suggesting sequencing errors and precluding a conclusive determination of genome modifications resulting from CRISPR targeting.

## Discussion

X-linked RP caused by *RPGR* mutations is an attractive target for gene therapy development because of its clinical severity and the large number of patients affected. It has been a subject of intensive investigations by many laboratories. Because the highly repetitive sequence in ORF15 has proved challenging to manipulate [20, 21, 37], current gene therapy approaches use various modified versions of *RPGR* to minimize inadvertent, spontaneous mutations in ORF15 while constructing and delivering expression vectors [13, 17, 18]. Several early phase trials are underway and preliminary results are encouraging [19]. However, efficacy is yet to be demonstrated and gene dosage will be a challenge for any strategy that relies on gene delivery, particularly if elevated protein levels show toxicity. Additional strategies, such as correction of the mutation in situ through genome editing are continuing to be explored as they offer appealing alternatives for patients with *RPGR* mutations.

In this study we investigated a simplified version of CRISPR/Cas9-mediated genome editing, taking advantage of several distinct features of the highly repetitive, purine-only region of the *RPGR* ORF15 exon. First, the length of this region can be altered without impacting protein function, therefore small deletions/insertions due to cell-mediated repair will be well tolerated. The differences in the length of this repetitive region suggest that it might not be playing an essential role in photoreceptors, and that restoration of the other protein domains such as the RCC1-like domain at the N-terminus and the basic domain at the C-terminus are providing the photoreceptors with enough activity to rescue the phenotype and delay retinal degeneration. Hence, the proposed gene editing strategy would have great potential, provided that the indels length that the NHEJ mechanism generates is not excessive. Second, a lack of pyrimidine residues means that stop codons in any of the reading frames will not be generated, thus minimizing the chances of translational termination prior to the shift back to the correct reading frame. Thus, the sole objective of correcting an *RPGR*^*ORF15*^ mutation is to shift the reading frame and restore expression of the C-terminal domain. The appeal of this design lies in bypassing template-dependent homologous recombination, a rate-limiting step in genome editing due to low efficiency that currently precludes its wide application in vivo. Our original design assumed the number of bases inserted or deleted during NHEJ would be nearly random, and thus up to one third of targeted cells could in theory have their ORF15 reading frame restored. We tested this design through both germline and somatic cell therapy approaches and confirmed that it was indeed possible to restore functional RPGR-ORF15 expression with this strategy. While the rescue was far from complete in the retina, this approach has a number of key advantages. The mutation was corrected in situ at the genome level which should remain stable. This contrasts with gene replacement therapy through delivery of extrachromosomal copies of vector DNA that relies on episomal persistence for lasting efficacy, which cannot be presumed. Furthermore, regulation of expression with our approach is controlled by native, cell intrinsic mechanisms instead of using heterologous promoter elements that could not fully replicate physiological expression profiles. Thus, in those cells that have had their *RPGR* expression restored, the treatment effect comes close to a “cure” and the efficacy should remain permanent.

This strategy, however, also has its limitations. It is not broadly applicable but could only be adopted to target mutations in genomic contexts similar to the *RPGR* gene. Because precise control over events that follow the initial DNA break is not feasible, the best-case scenario is having a portion of the target cell population rescued. While this is a major compromise, the redeeming features of this approach discussed above largely offsets the limitations in select instances, in comparison to current, state-of-the-art gene replacement strategies. As argued earlier, having a small portion of cells restored to a fully functional state would have a profoundly positive impact on the affected individuals and is thus a worthy pursuit.

Our study is the first to demonstrate template-free in vivo correction in mature photoreceptors as a proof-of-principle for future work that aims to correct disease-causing mutations in *RPGR*^*ORF15*^. While the finding that RPGR-ORF15 expression can be restored is encouraging, several questions remain that should be addressed in future studies. We were only able to achieve correction levels up to 10% of photoreceptors, which fell short of a theoretical 33% upper limit in the treated retinas. It should be noted that this theoretical upper limit was suggested on the assumption that the editing events would be largely mediated through NHEJ, and it would be only achievable upon dual transduction of both guide and Cas9 AAVs in 100% of the target cells, a condition unlikely to be met in real life situations. Furthermore, observations in the germline-targeted mice suggest that NHEJ did not appear to be the prevailing outcome in the present study (see below). Future efforts, therefore, should be directed at better understanding the molecular events following CRISPR-mediated DNA breaks in the RPGR gene and at improving overall AAV transduction efficiency to achieve a higher rate of gene correction. Recent reports [38] that NHEJ-associated base insertions/deletions are not random provide another possible explanation for the low gene editing rate. Interestingly, non-randomness in base insertions/deletions suggests that the process might be manipulated to achieve a higher gene correction rate than the theoretical limit. Other unanswered questions in this study relate to our inability to confirm the precise modifications introduced by CRISPR/Cas9 in the retina due to sequencing difficulties, a known issue with the ORF15 region of *RPGR*. Deep sequencing was performed on AAV-treated retinas to capture the range of gene modifications. Perhaps unsurprisingly, the ORF15 amplicon proved difficult to sequence, despite multiple attempts and platforms, and heterogeneity in the sequences obtained from both ORF15 targeted and control samples precluded confirmation of precise edits. Nevertheless, isoform-specific antibodies were used to show unambiguously that full-length RPGR-ORF15 protein was restored in *rd9* retina following treatment and that the protein was glutamylated and localized correctly to the connecting cilia. Finally, one of the main limitations of the CRISPR/Cas9 system is that deleterious off-target mutations can occur which could pose potential risks in clinical applications. Next-generation sequencing could detect such off-target mutations although sensitivity may be low when such mutations are rare. Prediction-based methods offer higher sensitivity of detection using target-specific DNA amplifications. These assays would help determine the safety profiles of the chosen CRISPR design. Because RPGR ORF15 sequences are poorly conserved between mice and humans, future choices of CRISPR guide RNA sequences will substantially differ as future studies transition to human cell-based models. In the current study in mice, we did not assess off-target effect of the CRISPR/Cas9 and cannot make any statement about off-targets of the CRISPR design in mice. It should be emphasized, however, that careful evaluation of off-target mutations, perhaps in human iPSC-derived retinal organoids, is critical to establish the safety profiles of this strategy.

An intriguing observation emerged from this study that suggests NHEJ may not be the only mechanism that mediates end joining and repair in the ORF15 exon. We were initially surprised to find that both founders born from independent batches of microinjections carried the same modification, which turned out to be a 32-bp deletion near the site of DNA breaks. We have conducted rigorous control experiments and evaluated breeding schemes ruling out any possibility that this could be due to cross contamination or mixing of mouse strains. Most tellingly, the founders showed restored RPGR^ORF15^ expression in patches of photoreceptors throughout their retinas which served as proof that these were genuinely chimeric founders. Moreover, the rate of transmission of the genome-edited allele was about 10–15%, which indicated a degree of genetic mosaicism as would be expected in the founders and was in sharp contrast with expected outcomes of contamination by wild type or heterozygous parental breeders. In view of the highly repetitive sequence context, our findings raised the intriguing possibility that deletion of the 32-bp segment was mediated by an alternative DNA repair mechanism such as microhomology-mediated end joining (MMEJ) [39, 40]. These results are consistent with the notion that the outcome of genome editing events is not random and instead can be related to intrinsic features of the target sequence [38, 41]. In the case of our current findings, we postulate that neighboring repetitive sequences within ORF15 may be used as a template for homologous recombination following a double-strand break in the DNA. This could happen because the ORF15 repetitive region is composed of a series of nearly identical, 32-bp repeats. Thus, in absence of exogenously provided DNA templates or sister chromatids, the break point could align to an adjacent 32-bp element localized in cis, through DNA looping, and to initiate recombination and repair. Completion of this process would then delete one 32-bp segment. This brings the ORF15 exon of *rd9* allele back to a correct reading frame and effectively rescues the disease-causing mutation. This hypothesis provides a plausible explanation for our unexpected finding in germline manipulated mice, and it would be very important to further validate this hypothesis in future experiments in mouse as well as human cells and tissues. As compared to NHEJ, an MMEJ driven process offers the exciting prospect that repair could be achieved at levels above the one third threshold, since the end joining would not be a random event. It would be important for future studies to take advantage of human iPSC-derived retinal organoids to test this approach on human mutations in *RPGR*^*ORF15*^. Such studies should ultimately determine if a simplified genome editing strategy as outlined in this work can become a legitimate future therapy for X-linked RP that is both safe and effective.
